# Honokiol Alleviates Methionine-Choline Deficient Diet-Induced Hepatic Steatosis and Oxidative Stress in C57BL/6 Mice by Regulating CFLAR-JNK Pathway

**DOI:** 10.1155/2020/2313641

**Published:** 2020-11-27

**Authors:** Ting Zhai, Wei Xu, Yayun Liu, Kun Qian, Yanling Xiong, Yong Chen

**Affiliations:** ^1^Hubei Province Key Laboratory of Biotechnology of Chinese Traditional Medicine, National & Local Joint Engineering Research Center of High-Throughput Drug Screening Technology, Hubei University, Wuhan 430062, China; ^2^Hubei Key Laboratory of Tumor Microenvironment and Immunotherapy, Medical College of China Three Gorges University, Yichang 443000, China

## Abstract

**Background:**

Honokiol (HNK) has been reported to possess various beneficial effects in the context of metabolic disorders, including fatty liver, insulin resistance, and oxidative stress which are closely related to nonalcoholic steatohepatitis (NASH), however with no particular reference to CFLAR or JNK.

**Methods:**

C57BL/6 mice were fed methionine-choline-deficient (MCD) diet and administered simultaneously with HNK (10 and 20 mg/kg once a day, ig) for 6 weeks, and NCTC1469 cells were pretreated, respectively, by oleic acid (OA, 0.5 mmol/L) plus palmitic acid (PA, 0.25 mmol/L) for 24 h, and adenovirus-down *Cflar* for 24 h, then exposed to HNK (10 and 20 *μ*mol/L) for 24 h. Commercial kits, H&E, MT, ORO staining, RT-qPCR, and Western blotting were used to detect the biomarkers, hepatic histological changes, and the expression of key genes involved in NASH.

**Results:**

The *in vivo* results showed that HNK suppressed the phosphorylation of JNK (pJNK) by activating CFLAR; enhanced the mRNA expression of lipid metabolism-related genes *Acox*, *Cpt1α*, *Fabp5*, *Gpat*, *Mttp*, *Pparα*, and *Scd-1*; and decreased the levels of hepatic TG, TC, and MDA, as well as the levels of serum ALT and AST. Additionally, HNK enhanced the protein expression of oxidative stress-related key regulatory gene NRF2 and the activities of antioxidases HO-1, CAT, and GSH-Px and decreased the protein levels of prooxidases CYP4A and CYP2E1. The *in vivo* effects of HNK on the expression of CLFAR, pJNK, and NRF2 were proved by the *in vitro* experiments. Moreover, HNK promoted the phosphorylation of IRS1 (pIRS1) in both tested cells and increased the uptake of fluorescent glucose 2-NBDG in OA- and PA-pretreated cells.

**Conclusions:**

HNK ameliorated NASH mainly by activating the CFLAR-JNK pathway, which not only alleviated fat deposition by promoting the efflux and *β*-oxidation of fatty acids in the liver but also attenuated hepatic oxidative damage and insulin resistance by upregulating the expression of NRF2 and pIRS1.

## 1. Introduction

Nonalcoholic fatty liver disease (NAFLD) is characterized by intracellular excess fat deposition and steatosis in liver histology without a history of excess alcohol consumption, which is similar to alcoholic liver disease [1]. According to the disease progress, the pathological changes of NAFLD are manifested as simple fatty liver, nonalcoholic steatohepatitis (NASH), liver fibrosis, cirrhosis, and ultimate liver cancer [2]. It is generally accepted that the “two hits” is the main pathogenesis of NAFLD/NASH. The theory considered that the first hit was an overaccumulation of lipids in the liver, while the second hit is based on the large amount of cytokines such as interleukin, tumor necrosis factor (TNF-*α*), and adiponectin, which cause oxidative stress and inflammatory response by participating in the metabolism of free fatty acids [3]. At present, the patients with NASH have amounted to hundreds of millions in the world. Although several kinds of drugs such as 6-ethylchenodeoxycholic acid have been proved in clinical trials to improve NASH with certain effect, but until now, there is still no clinical effective drugs for NASH [4]. Current treatments for NASH include lifestyle changes (such as dieting and enhancing physical exercise) [5], clinical application of antioxidant [6], insulin sensitizer [7], lipid-lowering drugs [8], and antidiabetic agents [9].

Honokiol (HNK) is a diphenyl compound derived from the traditional Chinese medicine *Magnolia officinalis*. It is usually used for antibacterial, anti-inflammatory, antivirus, and antitumor [10]. HNK can reduce fat deposition by inhibiting the expression of ACC, FAS, and SCD-1 which are the downstream genes of SREBP-1c in rats with an alcoholic fatty liver and in HepG2 cells treated by free fatty acids [11, 12]. In addition, HNK can significantly improve the hepatic steatosis of high-fat diet- (HFD-) induced mice [13], as well as the oxidative stress in type 2 diabetic rats fed HFD plus streptozotocin [14]. Our previous study showed that HNK decreased lipogenesis by inhibiting the expression of SREBP-1c and PNPLA3 and reduced lipid peroxidation by downregulating the protein levels of CYP2E1 and CYP4A in HepG2 cells with steatosis [15]. It was reported that caspase 8 and Fas-associated protein with death domain-like apoptosis regulator (CFLAR) could directly target ASK1 and interrupt its N-terminus-mediated dimerization, thereby blocking signaling involving ASK1 and JNK, subsequently improving hepatic lipid accumulation and IR by regulating PPAR*α* and IRS1, and improving inflammation by regulating c-Jun and c-Fos, which therefore regulates NASH in mice and nonhuman primates [16]. However, the effect of HNK on the CFLAR-JNK pathway associated with NASH remains unknown.

MCD diet is frequently used to establish a rodent model with NASH for exploring the hepatic lipid metabolic disorders and oxidative stress [17–21]. Oleic acid (OA) and palmitic acid (PA) treatment can lead to hepatocyte steatosis [22]. The present work was focused on the effect of HNK on the CFLAR-JNK pathway and its downstream key genes involved in lipid metabolism and oxidative stress in C57BL/6 mice fed MCD diet and in NCTC-1469 cells (normal liver cell line of mice) pretreated, respectively, by OA and PA and adenovirus-down *Cflar.*

## 2. Materials and Methods

### 2.1. Drugs and Reagents

The following are the drugs and reagents used in the study: HNK (lot number: 20131118, Wuhan Tai Kaisai, China); test kits for TG, TC, AST, ALT, CAT, GSH-Px, and MDA (Nanjing Jiancheng, China); test kit for intracellular TG content (Beijing Applygen, China); test kit for intracellular ROS content (Shanghai Beyotime, China); MCD diet (containing 175.7 g of methionine-choline free amino acid premix, 431.9 g of sucrose, 50 g of dextrin, 150.0 g of corn starch, 100.0 g of corn oil, 30.0 g of cellulose, and 52.4 g of mineral mix per 1000 g) and MCS diet (containing 175.7 g of methionine-choline free amino acid premix, 8 g of methionine, 2 g of choline chloride, 441.9 g of sucrose, 50 g of dextrin, 150.0 g of corn starch, 100.0 g of corn oil, 30.0 g of cellulose, and 52.4 g of mineral mix per 1000 g) (Nantong Trophy Feed Technology Co. Ltd., China); NCTC-1469 cells (donated by Wuhan University, China); OA (Aladdin, Shanghai, China); PA, MTT, and DMSO (Sigma, St. Louis, USA); 2-NBDG and trizol (Invitrogen, Carlsbad, USA); reverse transcriptase kit and real-time fluorescent quantitative PCR (RT-qPCR) kit (Toyobo-Shanghai Biotechnology Co. Ltd, China); antibodies including polyclonal rabbit anti-mice CFLAR, JNK, pJNK, IRS1, and NRF2 (Shenyang Wanlei, China); CYP2E1 (Wuhan Boster, China); CYP4A (Abcam, USA); polyclonal mice anti-mouse *β*-actin (Santa, USA); pIRS1 (Millipore, USA); horseradish peroxidase-labeled goat anti-rabbit IgG (H+L) used as the secondary antibodies of CFLAR, JNK, pJNK, NRF2, CYP2E1, and CYP4A (KPL, USA); horseradish peroxidase-labeled goat anti mice IgG (H+L) used as the secondary antibodies of *β*-actin (KPL, USA); and enhanced chemiluminescence liquid (ECL) (Shanghai Beyotime, China).

### 2.2. Animal Experiments

Six weeks of age male C57BL/6 mice (19 g-21 g) were supplied by the Disease Prevention and Control Center of Hubei Province (certificate number: SCXK 2011-0012). After acclimatization for one week, mice were kept in a controlled animal room at the condition of temperature (22 ± 2°C), 55%-65% humidity, and 12/12 h day/night cycle. Mice were randomly grouped into four groups and treated for 6 weeks as follows (*n* = 8 per group): Group 1 was fed a MCS diet; Group 2 was fed a MCD diet; and Groups 3 and 4 were fed a MCD diet and administered simultaneously with HNK (10 mg/kg or 20 mg/kg once a day, ig). Prior to execution by taking off the cervical spine, mice were fasted for 12 hours with no limit of water. Blood samples of all mice were obtained by extirpating eyeball. The liver was extract, weighed, and perfused with saline. One part of the liver was fixed in 4% paraformaldehyde for histopathological assays. The remaining part was exposed to ice water to prepare liver homogenate (liver saline, 1 : 9, *w*/*v*) for the biochemical indicator testing, RT-qPCR, and Western blotting. All experiments on mice were approved by the Ethics Committee for Scientific Research of Hubei University and were treated in compliance with the institutional guidelines for the care and use of laboratory animals.

The levels of AST and ALT in serum and TG, TC, MDA, CAT, GSH-Px, and HO-1 in the liver were examined by commercial kits according to the manufacturer's protocols.

The liver specimens of tested mice were fixed in 4% paraformaldehyde solution. After being embedded in paraffin and cut into 5 *μ*m thickness, the slices were stained with HE, ORO, and MT, respectively. The histological changes were observed using a light microscope to evaluate hepatic steatosis, inflammation, and fibrosis according to the reported method [23].

### 2.3. Cell Experiments

NCTC-1469 cells were cultured in DMEM containing 10% FBS and 100 U/mL penicillin and 100 U/mL streptomycin at 37°Cwith 5% CO_2_ and allowed to grow to 80% confluence. Cellular fatty degeneration was performed by pretreated NCTC-1469 cells with 0.25 mmol/L PA (dissolved in ultrapure water) plus 0.5 mmol/L OA (dissolved in methanol) for 24 h and adenovirus-down *Cflar* for 24 h, respectively. Then, the lipid-degenerated cells were treated with HNK (10 and 20 *μ*mol/L, dissolved in DMSO) for 24 h.

After the cells were pretreated by PA and OA, 1 × 10^6^ cells were inoculated into each well of a 6-well plate and divided into four groups: Group 1 was treated with cell culture medium (control group); Group 2 was pretreated with PA and OA (model group); and Groups 3 and 4 were pretreated with PA and OA, then treated with different concentrations of HNK (HNK groups).

To make adenovirus-mediated knockdown of *Cflar* in the cells, adenovirus was prepared as previously described [24], and short-hairpin RNA- (shRNA-) encoding DNA sequences were synthesized and constructed into adenovirus plasmids by Haisheng Biological Technology Co. Ltd. (Hangzhou, China). The shRNA sequences against *Cflar* were as follows: F-5′-GGGAAGAGTGTCTTGATGAAGATTCAAGAGATCTTCATCAAGACACTCTTCCTTTTTTG-3′ and R-5′-GATCCAAAAAAGGAAGAGTGTCTTGATGAAGATCTCTTGAATCTTCATCAAGACACTCTTCCCTGCA-3′. Cells were inoculated into a 6-well plate and divided into 4 groups: Group 1 was treated with adenoviruses expressing shRNA against luciferase (Ad-shCtrl) (control group); Group 2 was infected with adenovirus expressing shRNA against *Cflar* (Ad-sh*Cflar*) at 100 plaque-forming units (PFU)/cell (model group); and Groups 3 and 4 were pretreated with Ad-sh*Cflar*, then treated with different concentrations of HNK (HNK groups).

Cell cytotoxicity was examined via MTT assay. Cells were inoculated at a density of 1 × 10^3^ per well in 96-well plates and cultured with various concentrations (0–200 *μ*mol/L) of HNK (dissolved in DMSO) for 24, 48, and 72 h. 20 *μ*L MTT (5 mg/mL) was then added to each well, and cells were cultured for a further 4 h. Subsequently, 150 *μ*L DMSO was added to dissolve the precipitate. Finally, the absorbance at 570 nm was recorded using iMark microplate reader (Bio-Rad, USA).

The intracellular TG and ROS contents were detected using a TG quantification kit and ROS assay kit according to the manufacturer's instructions. To investigate the glucose uptake, NCTC-1469 cells inoculated in 6-well plate were cotreated with PA and OA for 24 h, then treated with HNK (10 and 20 *μ*mol/L) for 24 h, and finally were incubated in glucose-free DMEM with 2-NBDG (50 *μ*mol/L) for 30 min.

The fluorescent intensity of each well was measured using a multimode microplate reader (Berthold TriStar LB941, Germany) at excitation/emission wavelengths of 485 nm/535 nm. The protein concentration of each well was detected by BCA kit to normalize the data.

All of the *in vitro* cell experiments were performed independently in triplicate.

### 2.4. RT-qPCR

Total RNA from liver tissues (50-100 mg) and NCTC 1469 cells were extracted using trizol reagent (1 mL) plus chloroform (200 *μ*L). The bands of 28S, 18S, and 5S were detected by agarose gel electrophoresis for the integrity assessment of RNA. The quality of RNA samples was analyzed by a nucleic acid and protein analyzer (Eppendorf, Germany) at the absorbance of 260 nm and 280 nm. 3 *μ*g total RNA was reverse transcribed into cDNA using ReverTra Ace® qPCR RT kit (Toyobo, Japan). The levels of relative mRNA expression of the genes under test were analyzed by RT-qPCR using SYBR Green fluorescent quantitative PCR kit and CFX Connect™ Real-Time System (BIO-RAD, USA). The RT-qPCR were performed for 40 cycles at the following conditions: predenaturation at 95°C for 5 min, denaturation at 95°C for 30 s, and annealing at 58.3°C for 30 s and extended at 72°C for 30 s.  Table 1 shows the primer sequence of each tested gene.

### 2.5. Western Blotting

Total protein from liver tissues (0.1 g) and tested cells were, respectively, extracted in RIPA lysate containing protease and phosphatase inhibitors. 50 *μ*g total protein was used for SDS-PAGE, and the separated target protein was transferred to PVDF membrane. After being blocked at 25°C for 2 h, the membrane was, respectively, incubated with the primary antibodies of CFLAR, JNK, pJNK, IRS1, pIRS1, NRF2, CYP2E1, CYP4A, and *β*-actin at 4°C overnight and subsequently exposed to the appropriate secondary antibodies at 25°C for 1 h. Proteins were visualized by ECL and quantified by ImageJ software (US National Institutes of Health).

### 2.6. Statistical Analysis

Results were presented as the mean ± standard deviation (SD): *n* = 8 for the *in vivo* data and *n* = 3 for the *in vitro* data. Statistical analysis was performed using one-way analysis of variance (ANOVA), followed by Tukey's test. Differences between groups were considered to be statistically significant at *p* < 0.05.

## 3. Results

### 3.1. HNK Relieved MCD-Induced Hepatic Injury of Mice

The levels of serum ALT and AST, as well as hepatic TC and TG in the tested mice, are exhibited in Table 2. Compared with the MCS group, the levels of hepatic TC and TG and serum AST and ALT were significantly increased in the MCD group. After HNK treatment, the levels of serum AST and ALT, as well as hepatic TC and TG, were remarkably decreased compared with the MCD group. The *in vivo* effects of HNK on hepatic antioxidase activity and MDA content are also shown in Table 2. Contrasting to the MCS group, the activities of hepatic HO-1, CAT, and GSH-Px were obviously reduced, and the MDA level was significantly increased in the MCD group. However, HNK treatment remarkably increased the activities of these antioxidant enzymes and decreased the content of MDA compared with the MCD group.

The levels of ALT, AST, CAT, GSH-Px, and MDA in HNK groups were not back to those of the MCS group, but surprisingly, HO-1 activity of the HNK high-dose group was about 2 times compared to that of the MCS group. Anyway, the results indicated that HNK had a beneficial effect on MCD-induced hepatic injury and fat accumulation in mice.

**Figure 1:**
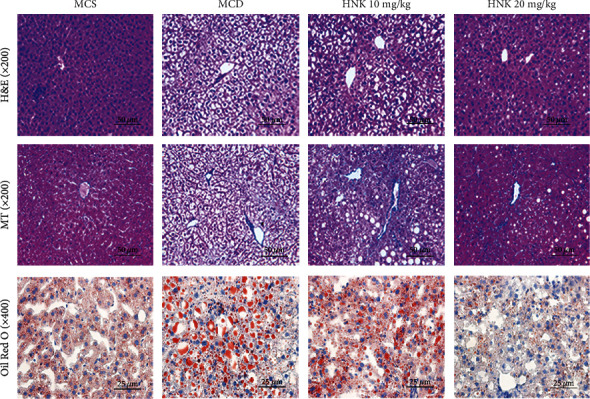
Effects of HNK on hepatic histological changes. H&E, MT, and ORO staining of liver sections in different groups of mice.

Figure 1 shows the histological changes including hepatic steatosis, ballooning, inflammation, and fibrosis in the tested mice which were examined by HE, ORO, and MT staining, respectively. Histological changes including steatosis, ballooning, and inflammation were observed in the MCD group according to the results from HE and ORO staining, whereas these changes were restored by HNK treatment. However, HNK treatment had no significant effect on hepatic fibrosis based on the results of MT staining.

### 3.2. HNK Upregulated the mRNA Expression of Lipid Metabolism-Related Genes In Vivo

**Figure 2:**
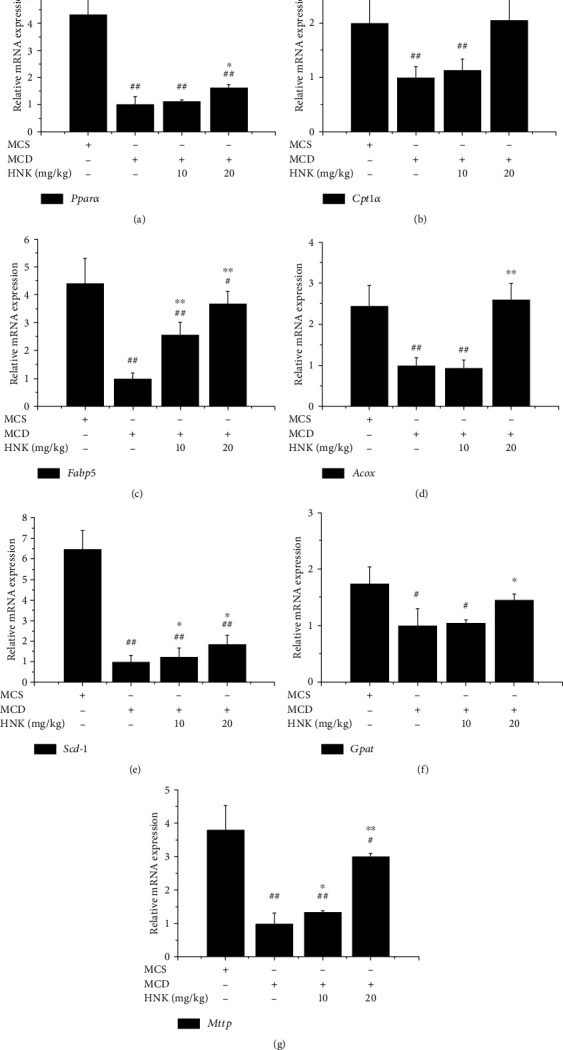
Effects of HNK on the mRNA expression of hepatic lipid metabolism-related genes of the tested mice. The mRNA expression was analyzed by RT-qPCR. *n* = 8. ^#^*p* < 0.05 and ^##^*p* < 0.01*vs.* MCS. ^∗^*p* < 0.05 and ^∗∗^*p* < 0.01*vs* MCD.

RT-qPCR was applied to examine the relative mRNA expression of lipid metabolism-related key genes in the liver of the tested mice, and the results are shown in Figure 2. Compared with the MCS group, MCD treatment downregulated the mRNA levels of *Pparα*, *Fabp5*, *Acox*, *Cpt1α*, *Scd-1*, *Gpat*, and *Mttp*, while HNK treatment markedly raised the mRNA levels of *Pparα*, *Fabp5*, and *Scd-1* at low dose (10 mg/kg), as well as *Pparα*, *Fabp5*, *Acox*, *Cpt1α*, *Scd-1*, and *Mttp* at high dose (20 mg/kg). Although the mRNA expression levels of the tested genes at low dose of HNK were significantly lower than those of the MCS group, the mRNA expression levels of *Acox* and *Cpt1α* had no significant difference between the MCS group and the HNK-high dose group.

### 3.3. HNK Regulated the Protein Expression of CFLAR-JNK Pathway-Related Genes In Vivo

**Figure 3:**
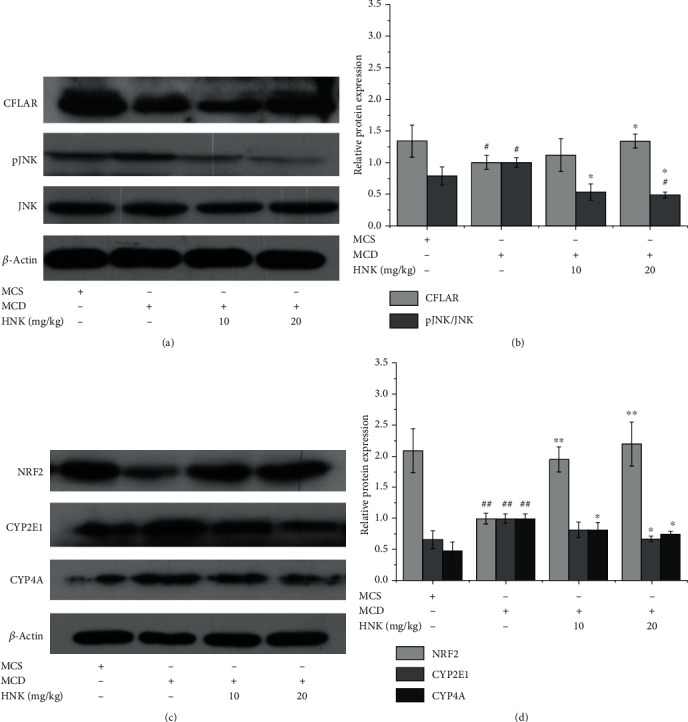
Effects of HNK on NASH-related protein expression in the liver of tested mice. The protein expression was analyzed by Western blotting. *n* = 8. ^#^*p* < 0.05 and ^##^*p* < 0.01*vs.* MCS. ^∗^*p* < 0.05 and ^∗∗^*p* < 0.01*vs.* MCD.

The effect of HNK on the protein expression levels of the hepatic CFLAR-JNK pathway-related genes including CFLAR, pJNK, NRF2, CYP2E1, and CYP4A in the tested mice was explored by using Western blotting, and the results are shown in Figure 3. Compared with the MCS group, the protein expression levels of CFLAR and NRF2 in the MCD group were significantly reduced, whereas the phosphorylation of JNK and the protein expression levels of CYP2E1 and CYP4A in the MCD group was significantly increased. HNK treatment reversed the observed changes induced by feeding MCD diet. Although HNK treatment could not return the protein levels of CYP2E1 and CYP4A to normal, the protein levels of CFLAR and NRF2 were restored to normal, and the level of pJNK/JNK was even lower than normal.

### 3.4. HNK Decreased the Contents of Intracellular TG and ROS and Promoted Glucose Uptake by Regulating the Expression of CFLAR-JNK Pathway-Related Genes in OA- and PA-Pretreated NCTC-1469 Cells

**Figure 4:**
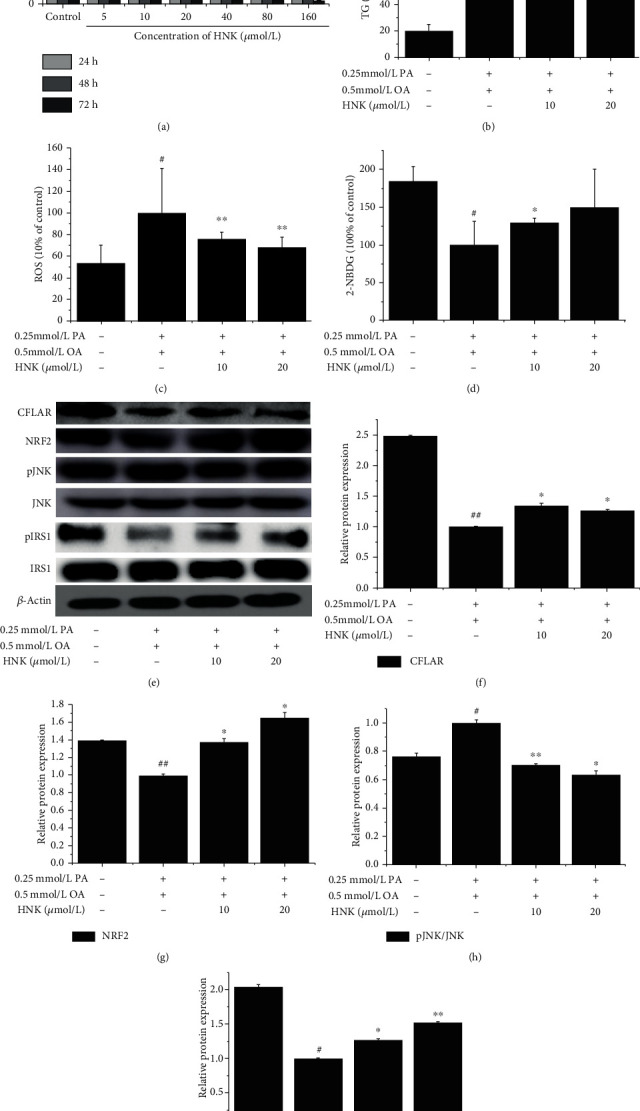
Effects of HNK on the cellular content of TG, ROS, and 2-NBDG and the protein expression of CFLAR, pJNK/JNK, NRF2, and pIRS1/IRS1 in OA- and PA-pretreated NCTC-1469 cells. NCTC-1469 cells were pretreated with OA and PA for 24 h, then treated with HNK for 24 h to detect the cellular viability (a) by MTT method, to detect the contents of TG (b), ROS (c), and 2-NBDG (d), and by the commercial kits, to detect the protein expression of CFLAR, NRF2, pJNK/JNK, and pIRS1/IRS1 (e)–(i) by Western blotting. *n* = 3. ^#^*p* < 0.05 and ^##^*p* < 0.01*vs.* the control group; ^∗^*p* < 0.05 and ^∗∗^*p* < 0.01*vs.* the model group.

The effects of HNK (0, 5, 10, 20, 40, 80, and 160 *μ*mol/L) on the viability of NCTC-1469 cells at 24, 48, and 72 h are shown in Figure 4(a). The NCTC-1469 cell viability was markedly reduced when treated with HNK at the concentrations of 40, 80, and 160 *μ*mol/L for 48 and 72 h, while HNK treatment at the concentrations of 10 and 20 *μ*mol/L for 24 to 72 h had no significant effect on the tested cell viability. So, 10 and 20 *μ*mol/L HNK were chosen to treat the cells for 24 h in the following experiments.

Figures 4(b)–4(d) show the effects of HNK treatment for 24 h on the contents of intracellular TG, ROS, and 2-NBDG, respectively. The contents of TG and ROS in OA- and PA-pretreated cells were obviously higher than those of control, and HNK treatment at 20 *μ*mol/L markedly decreased the levels of TG and ROS (Figures 4(b) and 4(c)). Additionally, OA and PA pretreatment suppressed the uptake of 2-NBDG in the tested cells, while HNK treatment dose dependently promoted the uptake of 2-NBDG in the tested cells (Figure 4(d)).

Figures 4(e)–4(i) show the effects of HNK on the protein expression of CFLAR, pJNK, NRF2, and phosphorylated IRS1 (pIRS1) in OA- and PA-pretreated cells. Compared with control, the protein levels of CFLAR, NRF2, and pIRS1 were markedly decreased, and the protein expression level of pJNK was markedly increased in OA- and PA-pretreated cells, whereas HNK treatment dose dependently reversed the above changes induced by OA and PA pretreatment, except the effect on CFLAR.

### 3.5. HNK Regulated the Expression of CFLAR-JNK Pathway-Related Genes in NCTC-1469 Cells Transfected with Ad-shCflar

**Figure 5:**
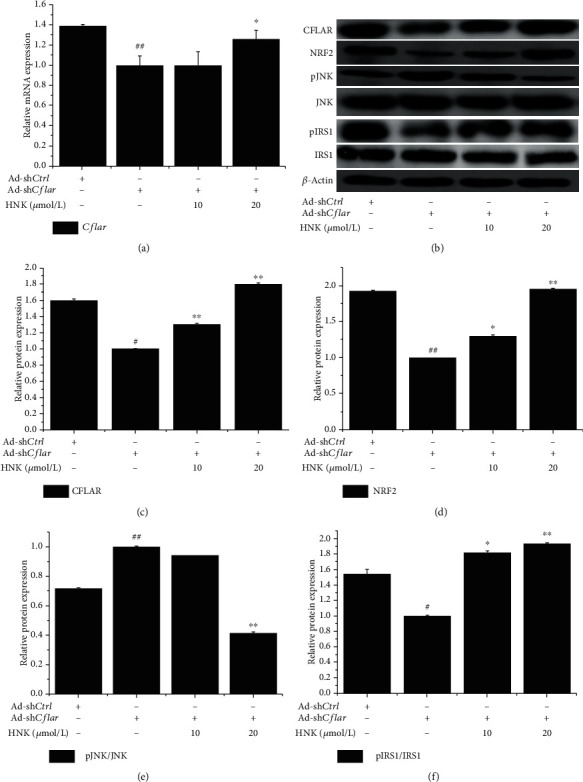
Effects of HNK on the expression of CFLAR-JNK pathway-related genes in NCTC-1469 cells transfected with adenovirus-mediated knockdown of *Cflar*. NCTC-1469 cells were transfected by Ad-sh*Cflar* for 24 h, then treated with HNK for 24 h to detect the mRNA and protein expression of CFLAR (a, b), as well as the protein expression of pJNK/JNK, NRF2, and pIRS1/IRS1 (c–f). The mRNA and protein expression was analyzed by RT-qPCR and Western blotting, respectively. *n* = 3. ^#^*p* < 0.05 and ^##^*p* < 0.01*vs.* the control group; ^∗^*p* < 0.05 and ^∗∗^*p* < 0.01*vs.* the model group.

To further confirm the effect of HNK on CFLAR-JNK pathway-related genes, NCTC-1469 cells were transfected with Ad-sh*Cflar* for 24 h and then treated with HNK for 24 h to detect the expression levels of CFLAR, NRF2, pJNK/JNK, and pIRS1/IRS1 in the tested cells. The results are shown in Figures 5(a)–5(f). Ad-sh*Cflar* treatment decreased the mRNA and protein expression of CFLAR (Figures 5(a) and 5(c)), as well as the protein expression of NRF2 (Figure 5(d)) and pIRS1/IRS1 (Figure 5(f)), while promoting the protein expression of pJNK/JNK (Figure 5(e)). HNK treatment upregulated the mRNA expression of *Cflar* (Figure 5(a)) and the protein levels of CFLAR (Figure 5(c)), NRF2 (Figure 5(d)), and pIRS1/IRS1 (Figure 5(f)), while downregulating the protein expression of pJNK/JNK in a dose-dependent manner (Figure 5(e)).

## 4. Discussion

The pathogenesis of NASH is complex, and its mainly clinical manifestations are hepatic lipid accumulation, oxidative stress, insulin resistance (IR), inflammatory responses, and fibrosis [25, 26]. CFLAR is the key regulator of the metabolic disorders associated with NASH, which can block the activation of apoptosis signal regulating kinase 1 (ASK1) by destroying ASK1 N-terminal mediated dimerization and affecting the phosphorylation of JNK and therefore alleviate NASH in mice and nonhuman primates [16]. It was reported that *Cflar* knockout obviously enhanced NASH-related pathological features of mice, whereas CFLAR overexpression significantly ameliorated NASH-related pathological changes of mice [16, 27]. Our study found that HNK treatment significantly increased the expression level of CFLAR and markedly decreased the phosphorylation of JNK in male C57BL/6 mice fed a MCD diet. Similar results were also found in NCTC1469 cells which were pretreated by OA plus PA and adenovirus-down *Cflar*, respectively. The effects of HNK on CFLAR-JNK pathway observed in this work are consistent with the previous reports [16, 27], and according to the previous research in the laboratory, the CFLAR-JNK pathway is closely related to the NASH [28], reflecting that HNK can inhibit the phosphorylation of JNK via activating CFLAR, ultimately alleviating the symptoms of NASH. To elucidate the mechanism of HNK against NASH, the present work focused on HNK-induced effects on the expression of downstream target genes of the CFLAR-JNK pathway involved in hepatic fat metabolism, oxidative stress, and IR.

Oxidative stress is closely related to NASH and observed in almost all patients with nonalcoholic fatty liver disease [29]. NRF2 is a highly sensitive transcription factor related to oxidative stress and plays a crucial regulation role in the gene expression of its downstream phase II detoxification enzymes, ubiquitin enzymes, antioxidases, and prooxidases [30, 31]. The expression of hepatic NRF2 was markedly downregulated in the MCD diet-induced NASH model; the activation of NRF2 significantly improved NASH-related symptoms [32]. CAT, GSH-Px, and HO-1 are important antioxidases for scavenging free radicals in organisms. CYP2E1 and CYP4A are important prooxidases for the generation of free radicals in the body. The expression and activity of hepatic CYP2E1 and CYP4A are not only closely related to hepatic oxidative stress but also strongly linked to the occurrence and development of NASH [33–35]. The protein expression of hepatic CYP2E1 and CYP4A in mice fed a MCD diet was remarkably upregulated, resulting in excessive free radicals that would aggravate lipid peroxidation of liver cells [36]. The present work found that the protein level of NRF2 and the activities of CAT, GSH-Px, and HO-1 were remarkably increased, whereas the protein levels of CYP2E1 and CYP4A were significantly decreased in the liver of mice fed a MCD diet following HNK treatment. Moreover, the *in vitro* effect of HNK on ROS content and NRF2 expression in hepatic cells also supports the *in vivo* effect of HNK on hepatic oxidative stress. The results indicated that HNK could alleviate NASH-related hepatic oxidative stress by increasing the antioxidase activity and inhibiting prooxidase activity.

IR is confirmed to be strongly associated with NASH. Insulin sensitivity of the muscle, liver, and adipose tissue was reduced in patients with NAFLD, along with the decreased glucose oxidation and glycogen synthesis [37–39]. IR can inhibit glucose uptake in hepatocytes [40] and can be improved by phosphorylation of IRS1 which is the downstream gene of JNK [41]. However, there is no insulin resistance in mice fed a MCD diet [42]. The present work found that HNK promoted fluorescent glucose uptake in OA- and PA-pretreated NCTC1469 cells and increased the phosphorylation of IRS1 in NCTC1469 cells which were pretreated by OA plus PA and adenovirus-down *Cflar*, respectively. Our results indicated that HNK could alleviate IR by upregulating pIRS1 in NCTC-1469 cells.

Many genes are associated with hepatic lipid metabolism, in which PPAR*α* plays a pivotal role in regulating the hepatic fat formation, the fibrosis of hepatic stellate cells (HSC), and the expression of its downstream target genes including ACOX, FABP5, and CPT1*α* [43]. ACOX is the first rate-limiting enzyme in the *β*-oxidation of fatty acids, and its expression increases the oxidation of fatty acids in the liver [44]. FABP5 is one of the fatty acid-binding protein families; due to its spatial structure, FABP5 can combine with the long chain fatty acids and transfer them to the target area such as mitochondria, endoplasmic reticulum, and nucleus, which further regulates the oxidation or esterification of fatty acids [45]. As a subtype of CPT1, CPT1*α* is one of the key enzymes for lipid metabolism, which controls the entry of free fatty acids (FFA) into mitochondria and their *β*-oxidation in the mitochondria [46]. The decreased expression of the three enzymes will affect the process of lipid transport, oxidation, and storage in hepatic cells and therefore lead to the accumulation of TC and TG in the liver. In addition, both SCD-1 and GPAT are the key enzymes for lipid synthesis and play an important regulatory effect on lipid balance in the body [47]. When the liver cells are exposed to excessive FFA, SCD-1 and GPAT can metabolize FFA to form TG; then, the downstream protein MTTP can further convert the lipids such as TG to chylomicron and very low-density lipoprotein (VLDL), make them secrete through the intestinal epithelial cells and liver cells, and eventually regulate the lipid balance in the body [48]. MCD diet was confirmed to induce hepatic fat deposition by decreasing the production of VLDL in the liver [49]. Meanwhile, the expressions of SCD-1, GPAT, and MTTP in the liver of mice with NASH were markedly inhibited [50, 51]. Our results showed that HNK significantly increased the mRNA expression of hepatic *Pparα*, *Fabp5*, *Cpt1α*, *Acox*, *Scd-1*, *Gpat*, and *Mttp* in mice with NASH, indicating that HNK could accelerate fatty acid *β*-oxidation by upregulating the mRNA expression of *Pparα* and its downstream target genes *Acox*, *Fabp5*, and *Cpt1α*; suppress fatty synthesis by increasing the mRNA expression of *Scd-1*, *Gpat*, and *Mttp*; and ultimately improve hepatic fat deposition.

In our study, adenovirus-down *Cflar of* NCTC1469 cells could decrease the protein expression of NRF2 and pIRS1/IRS1 and increase the protein expression of pJNK/JNK, while HNK could reverse the above effects. Unfortunately, there were no obvious changes on TG and ROS contents in adenovirus-down *Cflar* NCTC1469 cells. We suspected that the most possible reason was the lower knockdown efficiency of *Cflar* at 30%, which was not enough to affect the TG and ROS levels. This is the shortcoming of our study, and we will improve the experiment in the future study.

## 5. Conclusion

**Figure 6:**
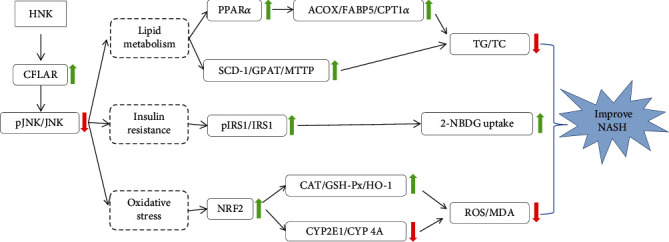
The proposed mechanism of HNK against NASH.

To sum up, HNK can inhibit the phosphorylation of JNK by activating CFLAR both *in vivo* and *in vitro*. The probable mechanism of HNK against NASH in mice is exhibited in Figure 6. Briefly, HNK not only relieved hepatic lipid accumulation by promoting the *β*-oxidation and efflux of fatty acids in liver but also improved hepatic oxidative stress by increasing the activities of antioxidases (CAT, GSH-Px, and HO-1) and decreasing the activities of prooxidases (CYP2E1 and CYP4A). Additionally, HNK alleviated hepatic IR by promoting the phosphorylation of IRS1 in NCTC1469 cells with steatosis.

## Figures and Tables

**Table 1:** Primer sequences used for RT-qPCR.

Gene	Primer	Sequence (5′⟶3′)
*β-Actin*	Forward	AACCGTGAAAAGATGACCCAGAT
	Reverse	CACAGCCTGGATGGCTACGTA
*Pparα*	Forward	CGGGAAAGACCAGCAACAAC
	Reverse	ATAGCAGCCACAAACAGGGA
*Fabp5*	Forward	GGAAGGAGAGCACGATAACAAGA
	Reverse	GGTGGCATTGTTCATGACACA
*Cpt1α*	Forward	TCCACCCTGAGGCATCTATT
	Reverse	ATGACCTCCTGGCATTCTCC
*Acox*	Forward	CGGAAGATACATCCCGGAGACC
	Reverse	AAGTAGGACACCATACCACCC
*Scd-1*	Forward	TACTACAAGCCCGGCCTCC
	Reverse	CAGCAGTACCAGGGCACCA
*Gpat*	Forward	CCATTGTGGAGGATGAAGTG
	Reverse	TGGATCGTGCCAGATAGGGA
*Mttp*	Forward	GCTAAGAAGCTGATAATGGGAGG
	Reverse	CCACTCTTGGAGAAACGGTCATA
*Cflar*	Forward	CTGTGTCTGCCGAGGTCATTC
	Reverse	AGAGCAATTCAGCCAAGGTAGC

**Table 2:** Effect of HNK on the hepatic injury-related biomarkers of the tested mice.

Biomarker	MCS	MCD	HNK (mg/kg)	
			10	20
Serum ALT (U/L)	8.59 ± 1.13	41.42 ± 9.38^##^	17.88 ± 6.39^##,^^∗∗^	12.76 ± 1.32^##,^^∗∗^
Serum AST (U/L)	21.37 ± 3.98	40.32 ± 6.23^##^	32.32 ± 7.52^##,^^∗^	31.94 ± 4.8^##,^^∗^
Hepatic TC (mmol/gprot)	0.09 ± 0.02	0.19 ± 0.04^##^	0.06 ± 0.02^∗∗^	0.05 ± 0.01^∗∗^
Hepatic TG (mmol/gprot)	0.17 ± 0.04	0.35 ± 0.05^##^	0.28 ± 0.05^##^	0.25 ± 0.05^##,^^∗^
Hepatic MDA (nmol/mgprot)	0.91 ± 0.24	6.15 ± 1.48^##^	3.92 ± 0.72^##,^^∗^	3.86 ± 0.99^##,^^∗^
Hepatic CAT (U/mgprot)	22.56 ± 2.42	8.98 ± 1.22^##^	22.56 ± 2.21^∗∗^	19.97 ± 2.75^∗∗^
Hepatic GSH-Px (U/mgprot)	324.39 ± 42.04	189.57 ± 23.68^##^	236.69 ± 44.55^##,^^∗^	320.86 ± 47.75^∗∗^
Hepatic HO-1 (ng/mL)	3.42 ± 0.41	1.13 ± 0.24^##^	5.03 ± 0.13^∗∗^	6.23 ± 0.10^∗∗^

*n* = 8. ^#^*p* < 0.05 and ^##^*p* < 0.01*vs.* the MCS group; ^∗^*p* < 0.05 and ^∗∗^*p* < 0.01*vs.* the MCD group.

## Data Availability

The data used to support the findings of this study are included within the article.

## Abbreviations

ACOX: Acyl-coenzyme A oxidase X
ALT: Alanine aminotransferase
AST: Aspartate aminotransferase
CAT: Catalase
CFLAR: Caspase 8 and Fas-associated protein with death domain-like apoptosis regulator
CPT1*α*: Carnitine palmitoyl transferase 1*α*
CYP2E1: Cytochrome P450 2E1
CYP4A: Cytochrome P450 4A
FABP5: Fatty acid-binding protein 5
GSH-Px: Glutathione peroxidase
GPAT: Glycerol-3-phosphate acyltransferase
HO-1: Heme oxygenase 1
IRS1: Insulin receptor substrate 1
JNK: c-Jun N-terminal kinase
HE: Hematoxylin-eosin
MCS: Methionine-choline sufficient
MCD: Methionine-choline deficient
MT: Masson trichrome
MTTP: Microsomal triglyceride transfer protein
MDA: Malondialdehyde
MTT: 3-[4,5-Dimethylthiazol-2-yl]-2,5-diphenyltetrazolium bromide
NASH: Nonalcoholic steatohepatitis
NAFLD: Nonalcoholic fatty liver disease
NRF2: Nuclear factor erythroid 2-related factor 2
ORO: Oil red O
OA: Oleic acid
PA: Palmitic acid
pJNK: Phosphorylation of c-Jun N-terminal kinase
pIRS1: Phosphorylation of insulin receptor substrate 1
PPAR*α*: Peroxisome proliferator-activated receptor *α*
ROS: Reactive oxygen species
SCD-1: Stearoyl-coenzyme A desaturase-1
TG: Triglyceride
TC: Total cholesterol
2-NBDG: 2-(N-(7-Nitrobenz-2-oxa-1,3-diazol-4-yl) amino)-2-deoxyglucose.
